# Optimization and Clinical Evaluation of a Multi-Target Loop-Mediated Isothermal Amplification Assay for the Detection of SARS-CoV-2 in Nasopharyngeal Samples

**DOI:** 10.3390/v13050940

**Published:** 2021-05-19

**Authors:** Foteini Roumani, Sarah Azinheiro, Hugo Sousa, Ana Sousa, Mafalda Timóteo, Tatiana Varandas, Daniela Fonseca-Silva, Inês Baldaque, Joana Carvalho, Marta Prado, Alejandro Garrido-Maestu

**Affiliations:** 1 Food Quality and Safety Research Group, International Iberian Nanotechnology Laboratory, Av. Mestre José Veiga s/n, 4715-330 Braga, Portugal; foteini.roumani@inl.int (F.R.); sarah.azinheiro@inl.int (S.A.); joana.carvalho@inl.int (J.C.); marta.prado@inl.int (M.P.); 2 Department of Analytical Chemistry, Nutrition and Food Science, School of Veterinary Sciences, University of Santiago de Compostela, Campus of Lugo, 27002 Lugo, Spain; 3 Virology Service, Portuguese Oncology Institute of Porto, Rua Dr. António Bernardino de Almeida, 4200-072 Porto, Portugal; hugo.sousa@ipoporto.min-saude.pt (H.S.); ana.saraiva.sousa@ipoporto.min-saude.pt (A.S.); tatiana.varandas@ipoporto.min-saude.pt (T.V.); daniela.freitas@ipoporto.min-saude.pt (D.F.-S.); Ibaldaque@ipoporto.min-saude.pt (I.B.); 4 Molecular Oncology and Viral Pathology Group (CI-IPOP), Portuguese Oncology Institute of Porto, Rua Dr. António Bernardino de Almeida, 4200-072 Porto, Portugal; m.timoteo@campus.fct.unl.pt

**Keywords:** SARS-CoV-2, RT-LAMP, clinical evaluation, ORF8, ORF3a, *N*

## Abstract

SARS-CoV-2 is the coronavirus responsible for COVID-19, which has spread worldwide, affecting more than 200 countries, infecting over 140 million people in one year. The gold standard to identify infected people is RT-qPCR, which is highly sensitive, but needs specialized equipment and trained personnel. The demand for these reagents has caused shortages in certain countries. Isothermal nucleic acid techniques, such as loop-mediated isothermal amplification (LAMP) have emerged as an alternative or as a complement to RT-qPCR. In this study, we developed and evaluated a multi-target RT-LAMP for the detection of SARS-CoV-2. The method was evaluated against an RT-qPCR in 152 clinical nasopharyngeal swab samples. The results obtained indicated that both assays presented a “good concordance” (Cohen’s k of 0.69), the RT-LAMP was highly specific (99%) but had lower sensitivity compared to the gold standard (63.3%). The calculated low sensitivity was associated with samples with very low viral load (RT-qPCR Cq values higher than 35) which may be associated with non-infectious individuals. If an internal Cq threshold below 35 was set, the sensitivity and Cohen’s k increased to 90.9% and 0.92, respectively. The interpretation of the Cohen’s k for this was “very good concordance”. The RT-LAMP is an attractive approach for frequent individual testing in decentralized setups.

## 1. Introduction

In late 2019, several cases of an atypical pneumonia were identified in China, which were linked to a novel coronavirus named as 2019 novel coronavirus, 2019-nCoV, and renamed as SARS-CoV-2 after identification of similarity to the severe acute respiratory syndrome (SARS) coronavirus. SARS-CoV-2 was then considered responsible for the coronavirus-associated disease (COVID-19). Its zoonotic origin was traced to bats due to its similarity with bat SARS-CoV-like coronaviruses, but it is speculated that an intermediate host also exists, at this moment pangolins being the most likely one [1]. At present, seven coronaviruses infecting human beings are known, of which four are associated with the common cold, and the other three (SARS-CoV, MERS-CoV, and SARS-CoV-2) are more pathogenic [2]. These viruses are enveloped, positive-sense, single-stranded RNA viruses, which belong to the genus *Betacoronavirus* [3].

The typical manifestations of COVID-19 are similar to those of SARS, which include fever, myalgia, dry cough, dyspnea, fatigue, and radiological evidence of ground-glass lung opacities compatible with atypical pneumoniae, but other symptoms differ, such as less frequent diarrhea compared to SARS [4]. COVID-19 has spread rapidly worldwide, having reported at the time of this study almost 160 million cases causing more than 3.3 million deaths in 221 countries (https://www.worldometers.info/coronavirus/, accessed on 2 May 2021).

Due to this rapid dissemination of the virus, it is critical to accurately identify infected individuals in order to set up appropriate measures to limit further spread of the virus and so limit the disease and associated complications. The gold standard to perform the clinical diagnosis of the virus relies on its detection by reverse transcriptase real-time polymerase chain reaction (RT-qPCR) in nasopharyngeal swabs, for which several official protocols have been made publicly available, such as those from the CDC in the USA, the Institute Pasteur in France, or Charité in Germany [5,6,7]. Additionally, to cope with increasing need, FDA granted “emergency use authorization” (EUA) to many different diagnostic tools, including RT-qPCR methods, sequencing, and isothermal assays (https://www.fda.gov/medical-devices/coronavirus-disease-2019-covid-19-emergency-use-authorizations-medical-devices/vitro-diagnostics-euas#individual-molecular). Even though all these methods are highly reliable and sensitive for the detection of SARS-CoV-2, as evidenced above, the high number of reported cases and the rapid spread have generated reagent shortages in certain locations, in particular, low-resource countries. This, in addition to the turnaround time needed to obtain an RT-qPCR result, has generated the need for the development of alternative methodologies suitable for population surveillance and fast clinical diagnosis. Among these, isothermal nucleic acid-based techniques have found a niche, and a plethora of methodologies have been reported recently [8,9,10,11]. Many of these novel methods are based on loop-mediated isothermal amplification (LAMP) using fluorescent dyes and probes [12,13], naked-eye color change observation [14,15,16], or even combining it with breakthrough technologies such as CRISPR-Cas and lateral flow [17,18,19,20,21,22].

In the current study, the main goal was to develop a multi-target reverse transcriptase LAMP (RT-LAMP) assay for the detection of SARS-CoV-2. The performance of the methodology was evaluated against a panel of randomly collected clinical nasopharyngeal swabs, which were gathered by the Virology Service of the Portuguese Oncology Institute of Porto (IPO-Porto), and the results were compared against their gold standard RT-qPCR methodology.

## 2. Materials and Methods

### 2.1. Viral Controls and Gene Screening

For safety reasons, all the experiments performed in the evaluation of the methodology were performed either with the heat-inactivated SARS-CoV-2 virus (strain 2019-nCoV/USA-WA1/2020. ATCC^®^ VR-1986HK^™^, LGC Standards S.L.U., Barcelona, Spain), or with the Twist Synthetic SARS-CoV-2 RNA controls 1 to 6, as well as 14 and 15 (GISAID names: Australia/VIC01/2020, Wuhan-Hu-1, Japan/Hu_DP_kng_19-020/2020, USA/TX1/2020, USA/MN2-MDH2/2020, USA/CA9/2020, England/205041766/2020 and England/MILK-9E05B3/2020, TWIST Bioscience, South San Francisco, CA, USA). The batch of heat-inactivated virus acquired was provided at a final concentration of 3.75 × 10^5^ genome copies/µL, while the synthetic controls were indicated to have 10^6^ copies/µL. The synthetic controls 14 and 15 corresponded to the variant of concern (VOC) B.1.1.7. A rat coronavirus was used as negative control (ATCC^®^ VR 1410^™^, LGC Standards S.L.U., Barcelona, Spain). RNA was extracted using the NucleoSpin Dx Virus kit (Macherey-Nagel, Düren, Germany) following the manufacturer’s instructions.

**Figure 1:**
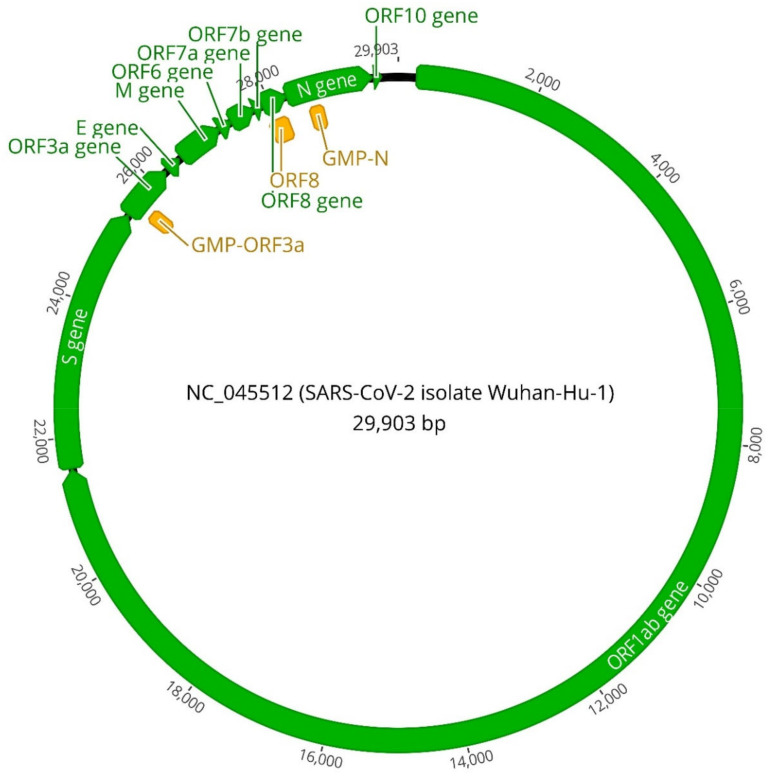
Circular representation of the complete genome of SARS-CoV-2 (NC_045512) corresponding to the isolate Wuhan-Hu-1, highlighting in green the different viral genes and in orange the fragments amplified by the primers selected in this study.

Due to its novelty and promising results, the primers described by Mautner et al., targeting the ORF8 of SARS-CoV-2, were selected to be used in this study [23]. In addition to these, new sets of primers were designed targeting the *N* gene and the ORF3a using primerexplorer V5 (http://primerexplorer.jp/lampv5e/index.html). Primer design was based on the sequences of SARS-CoV-2 NC_045512 for each gene, and was obtained from NCBI. Details regarding the specific sequences are provided in Table 1, and in Figure 1 the position of the selected genes, along with the actual amplicons generated by RT-LAMP, is represented.

### 2.2. Viral Target Evaluation

#### 2.2.1. RT-LAMP Reaction Setup

The setup of the reactions for all three genes was performed by fluorescent RT-LAMP with the following components: FIP/BIP 800 nM, F3/B3 200 nM, and LF/LB 400 nM, 12 µL of Fast Fluorescent mastermix (OptiGene Ltd., Horsham, UK), 0.4 µL of OptiRT (OptiGene Ltd., Horsham, UK), 0.04 µL of ROX reference dye (Invitrogen, Carlsbad, CA, USA), and 3 µL of template (final reaction volume 20 µL). Ten-fold dilutions from the heat-inactivated virus were prepared in RNAse/DNAse-free water (Thermo Fisher Scientific Inc., Waltham, MA, USA). The reactions were run at 65 °C for 30 min (the fluorescence data were acquired every 30 s over 60 cycles). The incubation was followed by a melt curve stage, which was performed by heating up the samples at 95 °C for 1 s, cooling down to 80 °C for 20 s, and heating again up to 95 °C, measuring the fluorescence over continuous increments of 0.015 °C/s. Having reached the final temperature, it was kept for 1 s. The reactions were performed in a QuantStudio 5 Real-Time PCR System with the QuantStudio™ Design and Analysis Software v1.4.3. (Applied Biosystems™, Foster City, CA, USA).

It is advised to avoid the opening of the reaction vessels, as LAMP is highly prone to false positive results due to amplification product cross-contamination. If this occurs, it will make it necessary to repeat the assays, thus delaying the results and increasing the cost of analysis [25,26,27].

#### 2.2.2. Determination of the Dynamic Range and Limit of Detection

The determination of the analytical sensitivity was performed using the synthetic control 1 as a reference. This control was ten-fold serially diluted from 10^6^ down to 50 copies. Each dilution was analyzed in triplicate for each one of the genes, and the test was repeated three times, thus rendering a total of nine replicates per concentration. The results were modelled by probit analysis with MedCalc (https://www.medcalc.org/).

### 2.3. Clinical Samples Processing and Reference Method

This study was approved by the Ethical Committee of IPO-Porto (232/020). Nasopharyngeal swab samples were collected from routine procedures for SARS-CoV-2 diagnosis at IPO Porto. After collection, samples were transported in sterile containers and delivered to the Virology Service in less than one hour, and stored at 4 °C until further processing. Samples were processed according to the safety instructions in a biosafety level 2 cabinet and inactivated with lysis buffer. The RNA extraction from clinical samples was performed with the reference protocol followed by the Virology Service of IPO-Porto, as detailed below.

The SARS-CoV-2 diagnostic at IPO-Porto was performed from 200 μL of sample with automated RNA extraction using the *Seegene^®^ StarMag Viral DNA/RNA kit* (Seegene Inc., Seoul, Republic of Korea) in a StarMAG System (Seegene Inc., Seoul, Republic of Korea), followed by RT-qPCR amplification with the *Allplex 2019-nCoV Assay* (Seegene Inc., Seoul, Republic of Korea) in a CFx-96 system from CFX96 Real-Time PCR Detection System (Bio-Rad, Feldkirchen, Germany). This assay targets four viral genes (*E*, *N*, and *S*/*RdRp*) and uses a total of 10 µL of template per sample. The results were analyzed using the Seegene Viewer software (Seegene Inc., Seoul, Republic of Korea), considering positive samples if amplification was observed for the targets at cycle threshold (Cq) < 40 as per manufacturer’s instructions.

### 2.4. Evaluation of the Methodology in a Clinical Setting

Nasopharyngeal swab samples collected at IPO-Porto were analyzed with the RT-LAMP assay and compared with the reference RT-qPCR protocol as previously described. With the RT-LAMP assay, a clinical sample was considered positive for SARS-CoV-2 if any of the selected genes were positive. The samples with a negative RT-LAMP, and with a delay in the internal control of the gold standard RT-qPCR, were tested for human *RNaseP* by LAMP to rule out reaction inhibition [24].

In this sense, those samples providing positive results by both methods will be categorized as positive agreements (PA); likewise, those with negative results by both assays will be considered negative agreements (NA). If the RT-LAMP method reported a positive result while the RT-qPCR was negative, it would be classified as a positive deviation (PD), and if the RT-LAMP obtained a negative result with a positive RT-qPCR, it will be considered a negative deviation (ND).

The categorized samples were used to calculate the diagnostic sensitivity, specificity, and accuracy (SE, SP, and AC, respectively) of the RT-LAMP method. In addition to these, the positive and negative predictive values (PPV and NPV) along with the Cohen’s kappa (k) were also calculated. These calculations were performed as previously published [28,29], and the 95% confidence interval (95% CI) was calculated as described in https://sample-size.net/.

## 3. Results and Discussion

### 3.1. Genetic Targets Performance

#### 3.1.1. RT-LAMP Reaction Setup

The three sets of primers correctly detected all the synthetic controls, along with the heat-inactivated human coronavirus, while no amplification was observed for the rat coronavirus. The specificity of the primers designed was further confirmed by BLAST analysis (https://blast.ncbi.nlm.nih.gov/Blast.cgi). Furthermore, the assay proved suitable for the detection of the VOC B.1.1.7. This variant is of great importance since it rapidly expanded in certain countries, and as of February 2021, accounted for 95% of the SARS-CoV-2 infections in England, and since then has been already identified in 82 countries [30]. All the selected sets of primers were capable of detecting this variant even though mutations were reported, namely in ORF8 and *N* [31].

**Figure 2:**
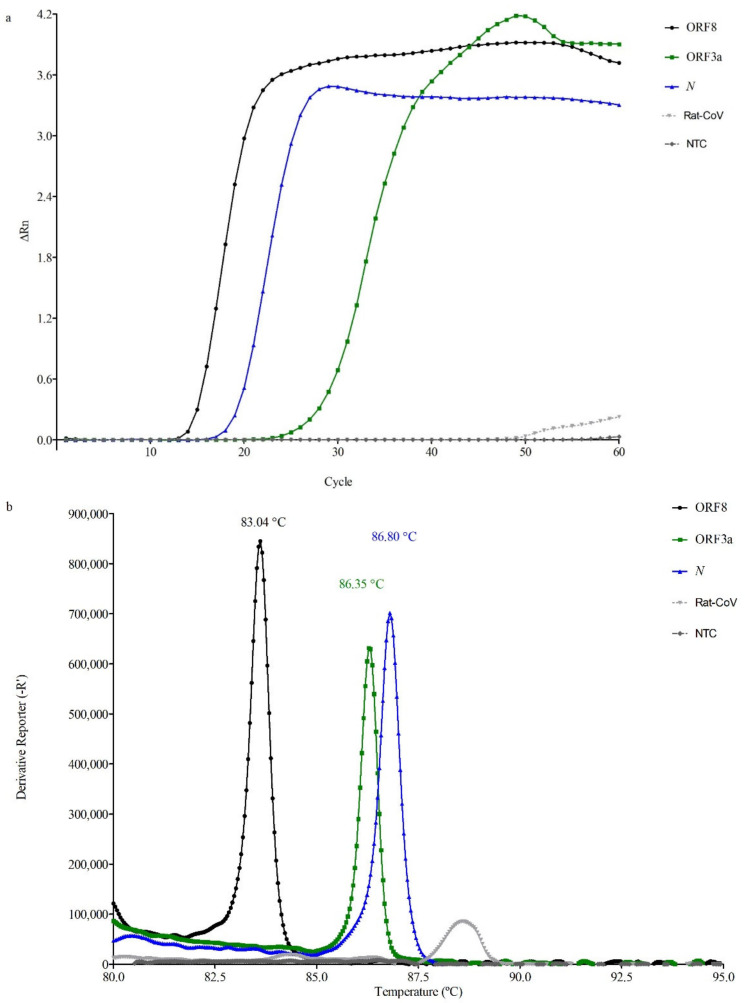
Typical amplification plots (**a**) and melt curves (**b**) obtained with the synthetic control 1 for ORF8, ORF3a, and *N*. ΔRn is the magnitude of normalized fluorescence signal, relative to the baseline fluorescence, generated by the reporter at each cycle during the amplification.

The gene reporting the fastest amplification was, on average, ORF8 as all the synthetic controls amplified in 5 to 8 min (average Tm 83.66 ± 0.17 °C), followed by *N* with results ranging from 7 to 10 min (Tm 86.88 ± 0.20 °C) and the slowest was the ORF3a, reporting positive results for these controls in 10 to 14 min (Tm 86.39 ± 0.20 °C). The Tm values with their corresponding SD were experimentally determined based on the results obtained for all the positive samples analyzed. Figure 2a,b show typical amplification plots along with their corresponding melt curves, which were generated by analyzing the synthetic control 1.

#### 3.1.2. Dynamic Range

**Figure 3:**
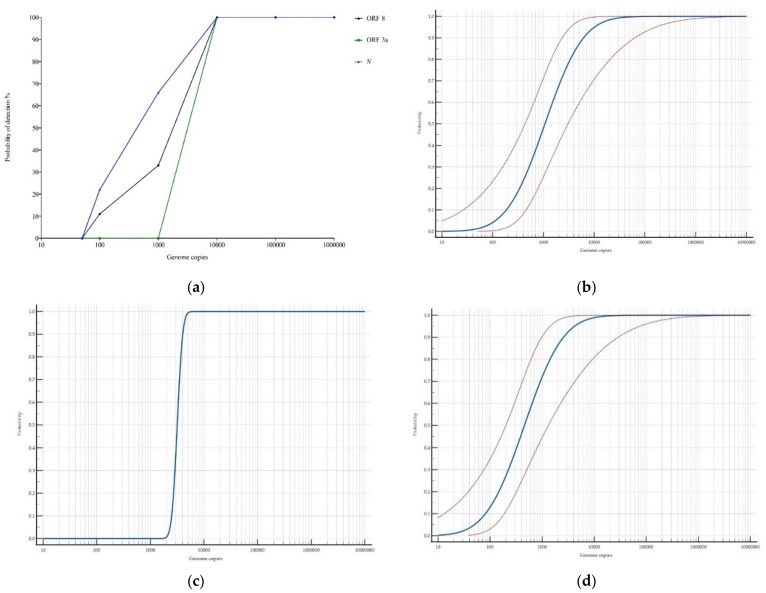
Detection probability of the fluorescence-based RT-LAMP for all the targets (**a**). The results of the probit analysis is shown in (**b**) for ORF8, (**c**) for ORF3a, and (**d**) for *N.* Graphs generated with MedCalc.

The analysis of the ten-fold dilutions of the synthetic control 1 indicated that all the targets were capable of reproducibly detecting the RNA down to 10^4^ copies, but differences became apparent below this quantity. With ORF3a, the different replicates of all the dilutions were negative below 10^4^ copies. Regarding ORF8, it was still possible to detect down to 100 copies but the detection probability dramatically decreased from 100% to 33% and 11% for the detection of 10^4^, 10^3^, and 100 copies, respectively. The most sensitive target was the *N* which was capable of detecting 10^3^ copies with a detection probability of 66% and 100 copies with a probability of 22%. With the current conditions, none of the targets were capable of detecting 50 copies. The results obtained are in the same range of other previously published RT-LAMP studies, such as the one from Jang et al., which reached 10^1^–10^2^ copies/µL targeting *RdRp*, *E*, and *N*, or that of Mautner et al., who reported limited detection of 10^2^ copies/µL [23,32]. Minor differences among assays may be explained due to the usage of different chemicals, primer concentration, and/or amplification temperature and time. The results are depicted in Figure 3a–d.

### 3.2. Clinical Samples Analyzed

**Figure 4:**
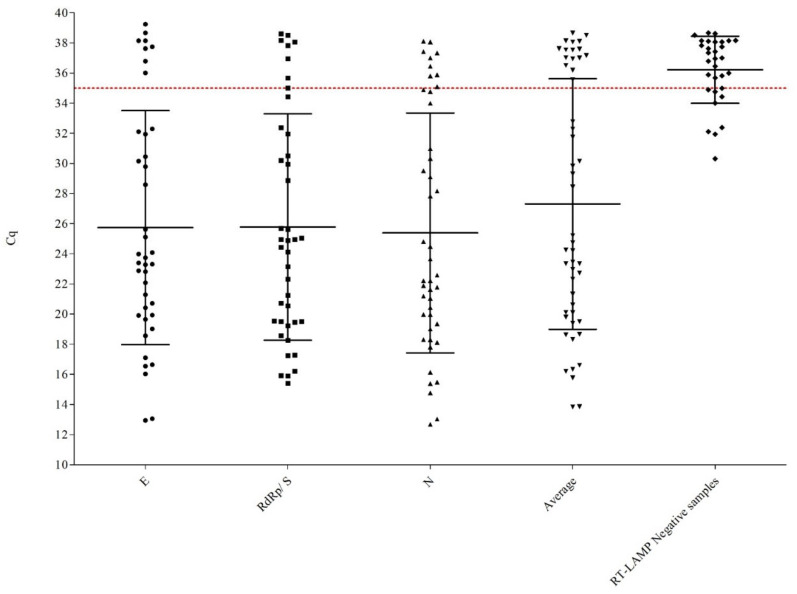
RT-qPCR Cq values obtained with the Allplex 2019-nCoV Assay for all the positive samples identified in this study. In each column, the average along with the standard deviation is preScheme 49. For positive samples identified by the reference RT-qPCR, 34 were positive for all the screened genes (*E*, *RdRp*/*S*, and *N*). One was positive for the combination *E- RdRP*/*S*, 5 for *RdRP*/*S*- *N*, 2 were positive for the combination *E*/*N*, and regarding samples with one single positive target by RT-qPCR, 1 sample was positive only for *RdRP*/*S,* 3 only for *N*, and another 3 only positive for *E*.

A total of 152 clinical samples were analyzed in the current study. Attending to the reference RT-qPCR protocol, 49 were positive and 103 were negative. Regarding the multi-target RT-LAMP assay, 31 were positive and 102 were negative, 1 sample was classified as a PD and 18 as ND. These results are summarized in Table 2 and in Figure 4 (specific Cq values obtained for each sample are provided in Table S1).

Regarding the RT-LAMP, out of the 31 positive samples identified, 21 were positive for the three targets, 5 were positive for the combination ORF8-*N*, and 5 were only positive for *N* (one of these 5 samples was negative by RT-qPCR and was considered a PD for the later evaluation). Overall, the highest number of positive samples was obtained targeting the *N* gene, but solely targeting this gene could result in detection problems when thinking on novel variants, which are constantly being reported; thus, targeting more than one gene/fragment would be highly recommended to avoid this issue [32]. In this regard, national regulations of different countries highlight the need for multiplex detection of positive result. Jang et al. also reported higher sensitivity targeting the *N* gene in their multiplex RT-LAMP assay implementing strand-displacement probes [32]. Some of the discrepancies between the RT-LAMP and the reference RT-qPCR assays may be explained by the difference in template analyzed (3 vs. 10 µL).

### 3.3. Clinical Evaluation

In the evaluation of the RT-LAMP methodology, only 1 PD was observed out of the 152 samples analyzed, thus demonstrating its high specificity, which was calculated to be of 99.0%. Regarding the sensitivity, the results were not as good since 18 ND were obtained, rendering a sensitivity of 63.3%. This resulted in an overall efficiency of the method of 87.5%, a positive predictive value of 96.9%, a negative predictive value of 85.0%, and a Cohen’s k of 0.69. Nevertheless, this is interpreted as “good concordance” between the novel and the reference method. Similar results were reported in other studies dealing with the evaluation of RT-LAMP assays for the rapid diagnosis of COVID-19 [34].

In Table 3, the percentage of positive results for each target gene per Cq value range is summarized. These results confirmed those obtained when evaluating the dynamic range, where the *N* gene was the most sensitive one followed by the ORF8 and lastly the ORF3a. Two samples with Cq values below 20 were not detected with the RT-LAMP for ORF8 and ORF3a, and one of these two was not detected by the *N* either. The sample which was negative for all the three genes by RT-LAMP was reanalyzed by RT-qPCR following the method described by the Institute Pasteur [5]. It was observed that the sample was now negative for all three targets (IP2, IP4, and *E*) along with the *S* gene designed in house (data not shown). In addition, due to shortage of RNA from the other misidentified sample, it was not possible to repeat the test (the sequence of the primers used in this confirmatory procedure is provided in Table S2).

It was observed that the low sensitivity of the assay was due to 18 misidentified samples. These samples were positive by RT-qPCR but negative by RT-LAMP. A closer look at these data highlighted the fact that the average Cq of these samples was 36.83 ± 1.81, see Figure 4. The high Cq value indicates that viral concentration in those samples was very low. Attending to previous reports, these patients may not even be infectious [35,36]. Only one exception was observed, and was associated with one single sample which had an average Cq of 18.62 ± 0.36 (19.01, 18.56, and 18.29 for *E*, *RdRp*/*S*, and *N*, respectively); however, the reported Cq value for the reference RT-qPCR internal control was 31.33 while this value tended to be in the range of 23–26 for the rest of the samples. However, LAMP analysis of the *RNaseP* gene did not show any significant effect of the RNA extract over the amplification; thus, this sample was finally classified as an ND (lack of amplification following the Institute Pasteur’s protocol is described above). In Figure 4, the individual Cq values for each sample are depicted, and the clustering of the samples being negative by RT-LAMP can be observed. These differences in sensitivity among RT-LAMP and RT-qPCR are in agreement with our own observations, as well as with those from other authors. Likewise, if the analysis of the results was limited to those samples with average Cq values below 35, as described in previous studies [9,34,37], only three samples would be classified as ND; thus, with this threshold set, the SP would remain at 99.0%, while the SE would increase up to 90.9%, the AC to 97.0%, the PPV and NPV to 96.8 and 97.1%, respectively, rendering a k of 0.92 that is interpreted as “very good concordance” among the reference and the alternative method [33]. The evaluation of the methodology focused on individuals with an active infection provided better results than those previously reported, where the analysis of a similar group of samples only provided sensitivity results of 72.7%, but it must be kept in mind that the type of clinical samples analyzed was not the same, saliva against nasopharyngeal swabs [35]. All the results of the evaluation are summarized in Table 2.

Overall, the performance of the reported methodology is similar to other previously published rapid molecular tests. Similar to what happened with the *Abbott ID Now COVID-19 test*, the limit of detection in clinical samples is higher than that of RT-qPCR [37]. The specific values obtained for each one of the parameters evaluated are greatly influenced by the samples considered in the analysis, so if a great number of specimens present low viral loads, this will result in a drop in the reported performance of the assay, but it may not be actually representing a true limitation of the assay, as those samples may no longer be infectious, or may even be false positive results if proper control measures are not taken when running the RT-qPCR [38]. The fact that samples with low viral content, Cq values higher than 35, were not detected by RT-LAMP is in agreement with what was previously reported for this technique. This also highlights the fact that, when considering new methodologies, the provided performance results must be evaluated cautiously as they may be influenced by the specific samples taken into consideration, and may not accurately reflect the performance of the assay. For instance, in the study of Huang et al., where only eight positive samples were analyzed, it is claimed that positive samples with RT-qPCR values of 36.64 were also detected by RT-LAMP; however, this corresponded to one single sample, and the reported Cq value was for one single target, while the other viral targets screened presented Cq values of 34.9 and 31.18. Thus, this methodology should be further evaluated in order to obtain a more accurate idea of its real performance [8]. Similar results were identified in other studies [9,18,39,40]. A similar limitation was detected for rapid antigen tests, which are more economical, rapid, and simple than RT-qPCR, but are less sensitive. If a closer look is taken into the details provided for the *Panbio COVID-19 Ag Rapid Test*, the reported assay takes longer (30 vs. 15 min) but provides higher sensitivity as the antigen test only reaches values above 90% in samples with Cq values lower than 30, while the current assay achieves these values with Cq values lower than 35. Likewise, in a study from Nagura-Ikeda et al., molecular assays based on RNA detection were reported to be more sensitive than rapid antigen tests in saliva samples as they detected viral RNA in 50.5 to 81.6% of their samples applying different techniques (RT-qPCR and RT-LAMP), while only 11.7% were positive with the rapid antigen test they selected [41]. It is worth mentioning that these types of tests are commonly recommended for symptomatic individuals, rather than general population one-time-point testing. Overseeing this issue, if properly applied, the proposed method, like the rapid antigen tests, could be used for frequent population testing as a surveillance tool, so that by increasing the frequency of testing, the sensitivity will no longer be a limitation [39,40]. In addition to this proposed application, LAMP has been reported to be more robust in terms of resistance to inhibitors [42]; thus, the proposed method may also be applied to the analysis of environmental samples such as sewage water, which was reported to be useful for early prediction, and tracking of the movement of the virus [43,44,45].

## 4. Conclusions

The selected three sets of LAMP primers demonstrated good performance for RT-LAMP detection of SARS-CoV2. The use of a fluorescent mastermix, which allowed us to perform result confirmation by melt curve analysis, further enhanced the specificity of the assay. The methodology was evaluated in a set of random nasopharyngeal clinical swab samples, indicating that they have an excellent specificity, and reaching sensitivity levels similar to those of other previously reported rapid nucleic acid-based assays alternative to RT-qPCR. Overall, the results indicate that the proposed method would be more suitable for frequent population testing in a constant surveillance mode, rather than as a clinical diagnostic tool.

## Figures and Tables

**Table 1:** LAMP primers.

Target	Primer	Sequence (5′->3′)	Reference
ORF8	ORF8-FIP	AGG ACA CGG GTC ATC AAC TAC AAG CTG CAT TTC ACC AAGAA	[23]
	ORF8-BIP	AGG AGC TAG AAA ATC AGC ACC TAT GGG TGA TTT AGA ACC AGC	
	ORF8-F3	ACT TGT CAC GCC TAA ACG	
	ORF8-B3	CTA CCC AAT TTA GGT TCC TGG	
	ORF8-LF	TGG TTG ATG TTG AGT ACA TGAC	
	ORF8-LB	AAT TGA ATT GTG CGT GGA TGAG	
ORF3a	GMP-ORF3a-FIP	GAA GCG CTCT GAA AAA CAG CAA GAA G-CCT CAC TCC CTT TCG GAT	This study
	GMP-ORF3a-BIP	CTA GCA CTC TCC AAG GGT GTT CAC-GAG CAA AAG GTG TGA GTA AAC TG	
	GMP-ORF3a-F3	CGC GCT ACT GCA ACG ATA C	
	GMP-ORF3a-B3	TTC AAG GCC AGC AGC AAC	
	GMP-ORF3a-LF	GTG CAA CGC CAA CAA TAA GCC	
*N*	GMP-N-FIP	AGA CGG CAT CAT ATG GGT TGC A *tttt* GCG GGT GCC AAT GTG ATC	This study
	GMP-N-BIP	TCT GGC CCA GTT CCT AGG TAG T *tttt* GAC GAA TTC GTG GTG GTG AC	
	GMP-N-F3	ATT GGC TAC TAC CGA AGA GCT	
	GMP-N-B3	AGG AAG TTG TAG CAC GAT TGC	
	GMP-N-LF	TAC CAT CTT GGA CTG AGA TCT TTC A	
	GMP-N-LB	ACT GAG GGA GCC TTG AAT ACA CCA	
*RNaseP*	FIP	GTG TGA CCC TGA AGA CTC GGT TTT AGC CAC TGA CTC GGA TC	[24]
	BIP	CCT CCG TGA TAT GGC TCT TCG TTT TTT TCT TAC ATG GCT CTG GTC	
	F3	TTG ATG AGC TGG AGC CA	
	B3	CAC CCT CAA TGC AGA GTC	
	LF	ATG TGG ATG GCT GAG TTG TT	
	LB	CAT GCT GAG TAC TGG ACC TC	

“tttt” is an FIP/BIP sequence linker.

**Table 2:** Clinical evaluation of the RT-LAMP.

RT–qPCR Limit *	PA	NA	PD	ND	SE (95% CI)	SP (95% CI)	AC (95% CI)	PPV (95% CI)	NPV (95% CI)	k
RT–LAMP	31	102	1	18	63.3 (48.3–76.6)	99.0 (94.7–100)	87.5 (81.2–82.3)	96.9 (83.8–99.9)	85.0 (77.3–90.9)	0.69
RT–LAMP Cq <20	11	102	1	1	91.7 (61.5–99.8)	99.0 (94.7–100)	98.3 (95.9–100)	91.7 (61.5–99.8)	99.0 (94.7–100)	0.91
RT–LAMP Cq <25	24	102	1	1	96.0 (79.6–99.9)	99.0 (94.7–100)	98.4 (94.5–99.8)	96.0 (79.6–99.9)	99.0 (94.7–100)	0.95
RT–LAMP Cq <30	28	102	1	1	96.6 (82.2–99.9)	99.0 (94.7–100)	98.5 (94.6–99.8)	96.6 (82.2–99.9)	99.0 (94.7–100)	0.96
RT–LAMP Cq <35 **	30	102	1	3	90.9 (75.7–98.1)	99.0 (94.7–100)	97.1 (92.6–99.2)	96.8 (83.3–99.9)	97.1 (91.9–99.4)	0.92

* Results obtained by RT–LAMP for samples at the given RT–qPCR Cq value. ** Internally established threshold. PA: positive agreement. PD: positive deviation. NA: negative agreement. ND: negative deviation. SE: relative sensitivity. SP: relative specificity. AC: relative accuracy. PPV: positive predictive value. NPV: negative predictive value. k: index kappa of concordance. Interpretation of *k*: 0.4–0.6 “moderate concordance”; 0.61–0.8 “good concordance”; 0.81–1.00 “very good concordance” according to [33].

**Table 3:** Percentage of positive results for the RT-LAMP targets in different Cq value ranges.

Cq (Number of Samples)	ORF8 *n* (%)	ORF3a *n* (%)	*N n* (%)
<20 (*n* = 12)	10 (83.3%) *	10 (83.3%)^*^	11 (91.7%)^*^
20–25 (*n* = 13)	12 (92.3%)	11 (84.6%)	13 (100%)
25–30 (*n* = 4)	3 (75%)	0 (0%)	4 (100%)
30–35 (*n* = 16)	1 (6.25%)	0 (0%)	2 (12.5%)
>35 (*n* = 16)	0 (0%)	0 (0%)	1 (6.25%)

Cq value corresponds to the average value obtained for the three targets, *E*, *RdRp*/*S*, and *N*, of the *Allplex 2019-nCoV Assay*. * By excluding the sample that obtained negative results in the RT-qPCR following the Institute Pasteur’s protocol, the % increases to 91.7 for ORF 8 and ORF3a, and to 100% for *N.*

## Data Availability

Not applicable.

## Supplementary Materials

The following are available online at https://www.mdpi.com/article/10.3390/v13050940/s1, Table S1. Detailed results of the nasopharyngeal swabs analyzed in the current study by Allplex 2019-nCoV and RT-LAMP; Table S2. Genes, primers, and probes used for in house RT-qPCR.
