# Supplementary material for: Optimization and Clinical Evaluation of a Multi-Target Loop-Mediated Isothermal Amplification Assay for the Detection of SARS-CoV-2 in Nasopharyngeal Samples
Source: Viruses. 2021 May 19;13(5):940. doi: 10.3390/v13050940 (PMC8161362; doi:10.3390/v13050940)
Supplement: Supplementary file 1 [file viruses-13-00940-s001.zip › viruses-1208826-supplementary.pdf]

Table S1. Detailed results of the nasopharyngeal swabs analyzed in the current study by Allplex 2019-nCoV and RT-LAMP

	RT-qPCR (Cq)				RT-LAMP		
**Sample**	***E* gene**	***RdRp*/*S* gene**	***N* gene**	**IC**	**ORF 8**	**ORF 3a**	**GMP *N***
1	13.06	15.41	13.03	24.41	+	+	+
2	12.95	15.91	12.68	24.17	+	+	+
3	16.03	15.89	15.39	23.24	+	+	+
4	16.54	17.27	14.77	23.71	+	+	+
5	16.64	16.21	16.13	24.11	+	+	+
6	17.10	17.23	15.47	23.85	+	+	+
7	18.56	18.26	18.11	24.54	+	+	+
8	19.01	18.56	18.29	31.33	-	-	-
9	N/A	19.50	17.80	23.01	+	+	+
10	19.64	19.53	19.00	25.18	+	+	+
11	19.93	19.22	19.35	26.03	-	-	+
12	19.9	19.50	19.96	24.54	+	+	+
13	20.71	21.25	18.30	24.02	+	+	+
14	20.41	19.46	20.43	22.62	+	+	+
15	21.28	20.55	19.97	24.41	+	+	+
16	22.07	20.71	21.18	22.97	+	+	+
17	22.87	22.32	21.77	23.06	+	+	+
18	23.39	23.14	21.62	24.94	+	+	+
19	23.73	24.11	21.03	24.49	+	+	+
20	23.27	24.89	21.88	25.38	+	+	+
21	22.81	25.04	22.20	25.63	+	+	+
22	23.31	24.43	22.58	24.64	+	+	+
23	23.98	24.96	23.65	24.09	+	+	+
24	25.61	24.95	22.20	25.53	-	-	+
25	24.08	25.62	24.48	25.70	+	-	+
26	25.10	25.70	24.81	25.23	+	-	+
27	28.58	28.87	27.83	24.51	+	-	+
28	29.78	29.96	28.16	28.96	+	-	+
29	30.15	30.20	29.10	24.61	-	-	+
30	30.44	30.50	29.51	24.59	+	-	+
31	32.29	31.97	30.98	24.11	-	-	+
32	32.10	34.43	30.32	26.75	-	-	-
33	31.94	32.37	34.00	25.02	-	-	-
34	36.78	35.00	34.89	22.93	-	-	-
35	36.00	37.83	34.76	25.98	-	-	-
36	N/A	35.68	37.34	24.37	-	-	-
37	N/A	38.07	35.81	22.80	-	-	-
38	N/A	N/A	37.00	25.99	-	-	-
39	N/A	38.17	35.89	24.17	-	-	-
40	39.24	N/A	35.10	23.99	-	-	+
41	N/A	38.61	36.45	24.30	-	-	-
42	38.14	36.96	N/A	23.86	-	-	-
43	37.75	N/A	37.42	23.72	-	-	-
44	37.62	N/A	N/A	24.35	-	-	-
45	N/A	N/A	38.07	24.06	-	-	-
46	N/A	N/A	38.11	26.15	-	-	-
47	38.14	N/A	N/A	24.14	-	-	-
48	N/A	38.51	N/A	24.18	-	-	-
49	38.66	N/A	N/A	24.00	-	-	-
50	N/A	N/A	N/A	26.68	-	-	-
51	N/A	N/A	N/A	26.88	-	-	-
52	N/A	N/A	N/A	33.52	-	-	-
53	N/A	N/A	N/A	25.84	-	-	-
54	N/A	N/A	N/A	25.92	-	-	-

55	N/A	N/A	N/A	26.29	-	-	-
56	N/A	N/A	N/A	33.87	-	-	-
57	N/A	N/A	N/A	26.22	-	-	-
58	N/A	N/A	N/A	25.40	-	-	-
59	N/A	N/A	N/A	25.60	-	-	-
60	N/A	N/A	N/A	26.13	-	-	-
61	N/A	N/A	N/A	25.72	-	-	-
62	N/A	N/A	N/A	25.96	-	-	-
63	N/A	N/A	N/A	26.05	-	-	-
64	N/A	N/A	N/A	26.40	-	-	-
65	N/A	N/A	N/A	26.14	-	-	-
66	N/A	N/A	N/A	26.27	-	-	-
67	N/A	N/A	N/A	26.33	-	-	-
68	N/A	N/A	N/A	26.59	-	-	-
69	N/A	N/A	N/A	26.53	-	-	-
70	N/A	N/A	N/A	26.56	-	-	-
71	N/A	N/A	N/A	27.21	-	-	-
72	N/A	N/A	N/A	25.97	-	-	-
73	N/A	N/A	N/A	25.93	-	-	-
74	N/A	N/A	N/A	26.14	-	-	-
75	N/A	N/A	N/A	25.96	-	-	-
76	N/A	N/A	N/A	25.58	-	-	-
77	N/A	N/A	N/A	26.59	-	-	-
78	N/A	N/A	N/A	26.34	-	-	-
79	N/A	N/A	N/A	25.52	-	-	-
80	N/A	N/A	N/A	26.91	-	-	-
81	N/A	N/A	N/A	26.19	-	-	-
82	N/A	N/A	N/A	25.73	-	-	-
83	N/A	N/A	N/A	25.65	-	-	-
84	N/A	N/A	N/A	26.39	-	-	-
85	N/A	N/A	N/A	32.69	-	-	-
86	N/A	N/A	N/A	26.79	-	-	-
87	N/A	N/A	N/A	26.44	-	-	-
88	N/A	N/A	N/A	26.50	-	-	-
89	N/A	N/A	N/A	26.58	-	-	-
90	N/A	N/A	N/A	27.16	-	-	-
91	N/A	N/A	N/A	26.18	-	-	-
92	N/A	N/A	N/A	27.44	-	-	-
93	N/A	N/A	N/A	27.25	-	-	-
94	N/A	N/A	N/A	24.81	-	-	-
95	N/A	N/A	N/A	24.70	-	-	-
96	N/A	N/A	N/A	24.62	-	-	-
97	N/A	N/A	N/A	25.08	-	-	-
98	N/A	N/A	N/A	24.78	-	-	-
99	N/A	N/A	N/A	24.56	-	-	-
100	N/A	N/A	N/A	24.77	-	-	+
101	N/A	N/A	N/A	25.57	-	-	-
102	N/A	N/A	N/A	24.49	-	-	-
103	N/A	N/A	N/A	24.76	-	-	-
104	N/A	N/A	N/A	25.37	-	-	-
105	N/A	N/A	N/A	25.33	-	-	-
106	N/A	N/A	N/A	24.86	-	-	-
107	N/A	N/A	N/A	29.86	-	-	-
108	N/A	N/A	N/A	24.95	-	-	-
109	N/A	N/A	N/A	24.95	-	-	-
110	N/A	N/A	N/A	24.59	-	-	-
111	N/A	N/A	N/A	30.45	-	-	-
112	N/A	N/A	N/A	25.13	-	-	-
113	N/A	N/A	N/A	24.79	-	-	-

114	N/A	N/A	N/A	25.06	-	-	-
115	N/A	N/A	N/A	30.77	-	-	-
116	N/A	N/A	N/A	25.67	-	-	-
117	N/A	N/A	N/A	26.85	-	-	-
118	N/A	N/A	N/A	24.11	-	-	-
119	N/A	N/A	N/A	23.94	-	-	-
120	N/A	N/A	N/A	24.18	-	-	-
121	N/A	N/A	N/A	24.04	-	-	-
122	N/A	N/A	N/A	23.94	-	-	-
123	N/A	N/A	N/A	24.01	-	-	-
124	N/A	N/A	N/A	30.50	-	-	-
125	N/A	N/A	N/A	23.90	-	-	-
126	N/A	N/A	N/A	N/A	-	-	-
127	N/A	N/A	N/A	23.95	-	-	-
128	N/A	N/A	N/A	24.48	-	-	-
129	N/A	N/A	N/A	23.97	-	-	-
130	N/A	N/A	N/A	24.38	-	-	-
131	N/A	N/A	N/A	24.06	-	-	-
132	N/A	N/A	N/A	24.00	-	-	-
133	N/A	N/A	N/A	23.94	-	-	-
134	N/A	N/A	N/A	24.88	-	-	-
135	N/A	N/A	N/A	23.97	-	-	-
136	N/A	N/A	N/A	24.09	-	-	-
137	N/A	N/A	N/A	23.95	-	-	-
138	N/A	N/A	N/A	24.13	-	-	-
139	N/A	N/A	N/A	24.17	-	-	-
140	N/A	N/A	N/A	24.12	-	-	-
141	N/A	N/A	N/A	24.88	-	-	-
142	N/A	N/A	N/A	24.14	-	-	-
143	N/A	N/A	N/A	24.84	-	-	-
144	N/A	N/A	N/A	24.21	-	-	-
145	N/A	N/A	N/A	24.31	-	-	-
146	N/A	N/A	N/A	24.43	-	-	-
147	N/A	N/A	N/A	24.79	-	-	-
148	N/A	N/A	N/A	24.32	-	-	-
149	N/A	N/A	N/A	28.84	-	-	-
150	N/A	N/A	N/A	24.64	-	-	-
151	N/A	N/A	N/A	24.39	-	-	-
152	N/A	N/A	N/A	24.46	-	-	-

N/A and "-" indicate "not detected". IC is the Internal Control of the RT-qPCR.

Table S2. Genes, primers and probes used for *in house* RT-qPCR.

Target	Primer	Sequence	Reference
RdRp gene / nCoV_IP2	nCoV_IP2-12669Fw	ATGAGCTTAGTCCTGTTG	
	nCoV_IP2-12759Rv	CTCCCTTTGTTGTGTTGT	
	nCoV_IP2-12696bProbe(+)	^{FAM}AGATGTCTTGTGCTGCCGGTA ^{IABkFQ}	[5]
RdRp gene / nCoV_IP4	nCoV_IP4-14059Fw	GGTAACTGGTATGATTTCG	
	nCoV_IP4-14146Rv	CTGGTCAAGGTTAATATAGG	
	nCoV_IP4-14084Probe(+)	^{HEX}TCATACAAACCACGCCAGG ^{IABkFQ}	
N	E_Sarbeco_F1	ACAGGTACGTTAATAGTTAA-TAGCGT	
	E_Sarbeco_R2	ATATTGCAGCAGTACGCACACA	[6]
	E_Sarbeco_P1	^{FAM}ACAC-TAGCCATCCTTACTGCGCTTCG ^{IABkFQ}	
S	nCoV-2S_134.750F	TCCTGGTGATTCTTCTTCAGGT	This project
	nCoV-2S_134.883R	GGTCAAGTGCACAGTCTACAGC	
	nCoV-2S_134.776P	$^{AT-TO550N}$CAGCTGGTGCTGCAGCTTATTA ^{IAbRQSp}	

Reactions performed in a final volume of 20 µL with 3 µL of RNA template using the One-step NZYSpeedy RT-qPCR Probe kit, ROX from NZYTech. Primers and probes concentrations used were as detailed in the protocol of the Institute Pasteur, regarding the *S* gene 400 nM primers and 200 nM probe, were used.
